# Spectrophotometric Methods for Simultaneous Determination of Oxytetracycline HCl and Flunixin Meglumine in Their Veterinary Pharmaceutical Formulation

**DOI:** 10.1155/2017/2321572

**Published:** 2017-07-24

**Authors:** Hanan A. Merey, Mahmmoud S. Abd-Elmonem, Hagar N. Nazlawy, Hala E. Zaazaa

**Affiliations:** ^1^Analytical Chemistry Department, Faculty of Pharmacy, Cairo University, Kasr El Aini, Cairo 11562, Egypt; ^2^National Organization for Drug Control and Research (NODCAR), 6 Abu Hazem Street, Pyramids Ave., P.O. Box 29, Giza, Egypt

## Abstract

Four precise, accurate, selective, and sensitive UV-spectrophotometric methods were developed and validated for the simultaneous determination of a binary mixture of Oxytetracycline HCl (OXY) and Flunixin Meglumine (FLU). The first method, dual wavelength (DW), depends on measuring the difference in absorbance (ΔA 273.4–327 nm) for the determination of OXY where FLU is zero while FLU is determined at ΔA 251.7–275.7 nm. The second method, first-derivative spectrophotometric method (1D), depends on measuring the peak amplitude of the first derivative selectively at 377 and 266.7 nm for the determination of OXY and FLU, respectively. The third method, ratio difference method, depends on the difference in amplitudes of the ratio spectra at ΔP 286.5–324.8 nm and ΔP 249.6–286.3 nm for the determination of OXY and FLU, respectively. The fourth method, first derivative of ratio spectra method (1DD), depends on measuring the amplitude peak to peak of the first derivative of ratio spectra at 296.7 to 369 nm and 259.1 to 304.7 nm for the determination of OXY and FLU, respectively. Different factors affecting the applied spectrophotometric methods were studied. The proposed methods were validated according to ICH guidelines. Satisfactory results were obtained for determination of both drugs in laboratory prepared mixture and pharmaceutical dosage form. The developed methods are compared favourably with the official ones.

## 1. Introduction

**Figure 1:**
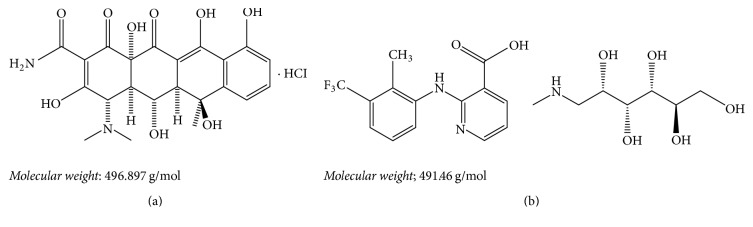
Chemical structures of (a) Oxytetracycline HCl and (b) Flunixin Meglumine.

Oxytetracycline HCl (OXY) (Figure 1(a)) is chemically designated as 4S,4aR,5S,5aR,6S,12aS-4-dimethylamino-1,4,4a,5,5a,6,11,12a octahydro-3,5,6,10,12,12a-hexahydroxy-6-methylene-1,11-dioxonaphthacene-2-carboxamide,5*β*-hydroxytetracycline as hydrochloride salt. It has a broad spectrum antimicrobial activity. It binds reversibly with 30S subunit of ribosome preventing the binding of aminoacyl transfer RNA and inhibiting proteins synthesis and so cell growth [1].

Flunixin Meglumine (FLU) (Figure 1(b)) is chemically designated as (2-[[2-methyl-3-(trifluoromethyl)-phenyl]amino]-3-pyridinecarboxylic acid compounded with 1-deoxy-1-(methylamino)-D-glucitol (meglumine salt). It is an NSAIDS used in veterinary medicine to relieve pain and inflammation in acute and chronic disorders [1]. It blocks some part of the cyclooxygenase enzyme pathway and thereby suppresses the synthesis of several chemical mediators of inflammation [2].

The mixture of OXY and FLU is formulated as injectable solution FLOXON® which is widely used as a veterinary drug indicated for the treatment of infectious diseases where concurrent analgesic, anti-inflammatory therapy is desired [3].

Oxytetracycline HCl and Flunixin Meglumine are official drugs in British Pharmacopeia [4], United State Pharmacopoeia [5], and European Pharmacopoeia [6].

Literature survey represented that several methods have been reported for determination of OXY alone or in combination with other drugs. These include spectrophotometric method [7], HPLC [8–11], colorimetric [12, 13], fluorometric [14, 15], and electrochemical methods [16], while FLU was determined by spectrophotometric [17], HPLC [18–23], and voltammetric methods [24, 25]. To the best of our knowledge, no method has been reported for the simultaneous determination of OXY and FLU in their pharmaceutical preparation.

Spectrophotometry has been widely accepted and extensively used in pharmaceutical analysis as an alternative to high performance liquid chromatography due to its simplicity, low coast, and short turnaround time.

The aim of this work was to develop different simple, sensitive, accurate, fast, and low-cost spectrophotometric methods, namely, dual wavelength, first derivative, derivative ratio, and ratio difference spectrophotometric methods capable of simultaneous determination of the aforementioned drugs in their dosage form. Different factors affecting each of the applied spectrophotometric methods were considered and experimental conditions were optimized.

## 2. Experimental

### 2.1. Apparatus

Shimadzu UV-2450 PC Series Spectrophotometer (Tokyo, Japan) with two matched 1 cm quartz cells using the following spectral parameters, a single fast scan mode and a slit width (2 nm), connected to a computer loaded with Shimadzu UV-PC software and used for all the absorbance measurements and data manipulation.

### 2.2. Pure Samples

OXY standard was kindly supplied by Farmachem SA, Mendrisio, Switzerland, and its purity was found to be 99.16 ± 0.896% according to the reported method [26], and FLU was supplied by Norbrook Lab. Ltd., Northern Ireland, UK. Its purity was found to be 99.84 ± 0.891% according to the official method [5].

### 2.3. Pharmaceutical Formulations

FLOXON injectable solution, labelled to contain 108 mg/mL of OXY and 33 mg/mL of FLU, batch number 160142, is manufactured by Pharma Swede Company [27]. It was purchased from local Egyptian market.

### 2.4. Reagents

All chemicals and solvents used were of analytical grade, hydrochloric acid, ADWIC (Cairo, Egypt); water used was distilled.

### 2.5. Standard Solutions

OXY and FLU standard stock solutions are 100 *µ*g/mL in 0.1 N HCl. They are stored in refrigerator whenever they are not used (they are stable for 14 days).

### 2.6. Laboratory Prepared Mixtures Containing Different Ratios of OXY and FLU

Into a series of 10 mL volumetric flasks, different aliquots OXY and FLU were transferred from their corresponding standard stock solutions (100 *µ*g/mL) of each, and then the volume was completed with 0.1 N HCl.

## 3. Procedures

### 3.1. Linearity

Aliquots equivalent to 50–600 *µ*g of OXY and FLU were accurately transferred from their respective standard stock solutions (100 *µ*g/mL) into sets of 10 mL volumetric flasks. The volume was completed to the mark with 0.1 N HCl. The zero-order absorption spectra of the prepared standard solutions were scanned from 200 to 400 nm under the previously mentioned spectral parameters and stored.

- * Dual Wavelength Method*. Linear relationships were directly constructed from zero-order spectra relating the difference in absorbance at ΔA_273.4–327 nm_ and ΔA_251.7–275.7 nm_ to the corresponding concentration of OXY and FLU, respectively.
- * First-Derivative Spectrophotometric Method*. The first derivative of the stored spectra of OXY and FLU was manipulated at Δ*λ* = 8 nm and scaling factor = 10. Calibration graphs were constructed relating the amplitude of the obtained first-derivative spectra at 377 nm and 266.7 nm to the corresponding concentrations of OXY and FLU (zero contribution of FLU and zero crossing of OXY), respectively.
- * Ratio Difference Spectrophotometric Method*. Ratio spectra of OXY and FLU were obtained by dividing the stored zero-order absorption spectra of OXY and FLU by the normalized divisor of FLU and OXY for the determination of OXY and FLU, respectively. Calibration graphs were constructed relating the difference in amplitudes of the ratio spectra at ΔP_286.5–324.8 nm_ and ΔP_249.6–286.3 nm_ to the determination of OXY and FLU, respectively.
- * First Derivative of Ratio Spectra*^*1*^*DD Spectrophotometric Method*. The first derivatives of the previously ratio spectra were obtained using Δ*λ* = 8 nm and scaling factor = 10. Calibration graphs were constructed relating peak to peak amplitudes of first derivative of the ratio spectra at P_296.7 nm + 369 nm_ and P_259.1 nm + 304.7 nm_ to the corresponding concentrations of OXY and FLU, respectively.
- The regression equations were then computed relating the response in each method to corresponding concentrations.

### 3.2. Analysis of Laboratory Prepared Mixtures

The absorption spectra of laboratory prepared mixtures were scanned and stored. Then procedures were performed as described under linearity and the concentrations of OXY and FLU in the prepared mixtures were obtained from the corresponding regression equation of each method.

### 3.3. Application to Pharmaceutical Formulation

One mL FLOXON solution (equivalent to 108 mg and 33 mg of OXY and FLU, resp.) was accurately transferred into 100 mL measuring flask; the volume was completed to the mark with 0.1 N HCl.

Suitable dilution was done using 0.1 N HCl to prepare a solution of final concentration equal to 27 *µ*g/mL and 8.25 *µ*g/mL of OXY and FLU, respectively. The procedures under linearity were followed to determine the concentrations of both drugs from the corresponding regression equation of each method.

## 4. Results and Discussion

**Figure 2:**
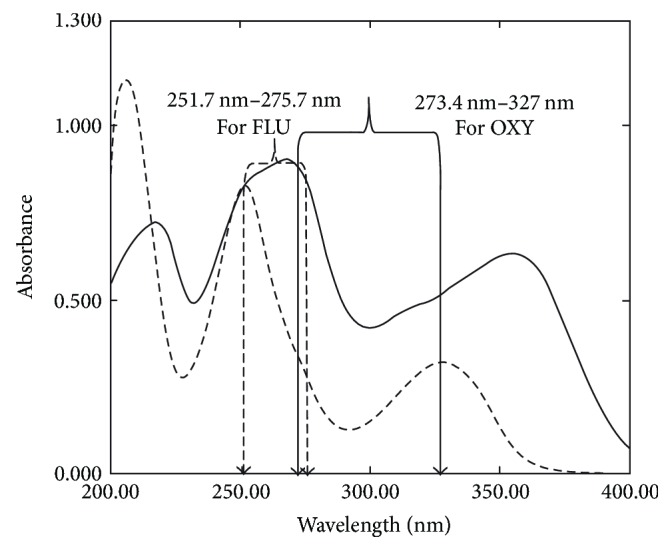
Zero-order spectra of 25 *µ*g/mL OXY (—) and 25 *µ*g/mL FLU (- - - - -) using 0.1 N HCl as a blank.

OXY and FLU are coformulated in FLOXON injection that is widely used as a veterinary dosage form indicated for the treatment of infectious diseases where concurrent analgesic, anti-inflammatory therapy is desired [3]. No publications were reported for the simultaneous determination of OXY and FLU in their pharmaceutical formulation; this acquire our attention to develop simple, accurate, and precise spectrophotometric methods for the simultaneous determination of both drugs without any preliminary separation to be used in quality control laboratories. Reviewing the structures of OXY and FLU illustrated that both drugs have different functional group affected by pH (pKa = 2.84 and 1.88 for OXY and FLU, res.), thus shifting the spectra of both drugs. Therefore different solvents were tried with different pH (water-NaOH-HCl-methanol); the best spectra of both drugs are obtained when using 0.1 N HCl as a solvent regarding the selectivity and reproducibility. The zero-order absorption spectra of OXY and FLU show severe overlapping between OXY and FLU (Figure 2) which prevents their direct determination. Therefore, we started with the simplest spectrophotometric method which is dual wavelength method. It is used to determine binary mixtures with overlapped spectra, as the difference in absorbance between two wavelengths is proportional to the absorbance of one component and zero contribution of the other component in the mixture [28–30]. For the determination of OXY, the difference in absorbance at 273.4 and 327 nm was selected where the difference in absorbance of FLU, at the same wavelengths, equals zero. Depending on the same principle, FLU could be determined by measuring the difference in absorbance at 251.7 and 275.7 nm where the absorbance difference of OXY at these two wavelengths equals zero (Figure 2). Linear relationships were obtained between the absorbance difference and the corresponding drug concentrations in the range of 5–60 *µ*g/mL for OXY and FLU.

**Figure 3:**
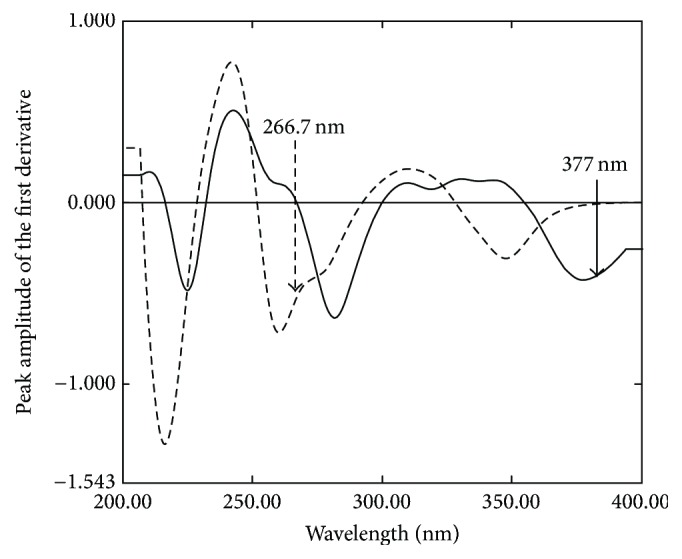
First-derivative absorption spectra of 60 *µ*g/mL OXY (—) and 60 *µ*g/mL FLU (- - - - - -) using 0.1 N HCl as a blank.

Derivative spectrophotometry is a valuable technique for resolving overlapped spectra of the binary mixture and for eliminating the effect of baseline shifts [30–32]. So, the first-derivative spectrophotometric method was tried to solve the overlapped spectra of OXY and FLU. The main parameters that affect the shape of the derivative spectra such as wavelength, scanning speed, and the wavelength increment over which the derivative is calculated (Δ*λ*) were studied. It was found that fast scanning speed, Δ*λ* = 8, and scaling factor 10 gave the best compromise in terms of signals to noise ratio, peak resolution, and sensitivity throughout the determination. By viewing the first-derivative spectra of OXY and FLU we found that OXY can be selectively determined at 377 nm where FLU has zero contribution, while FLU can be selectively determined at 266.7 nm as OXY shows zero crossing (Figure 3). Linear relationships were obtained between the peak amplitude of the first-derivative spectra at the selected wavelength and the corresponding drug concentrations in the range of 5–60 *µ*g/mL for OXY and FLU.

**Figure 4:**
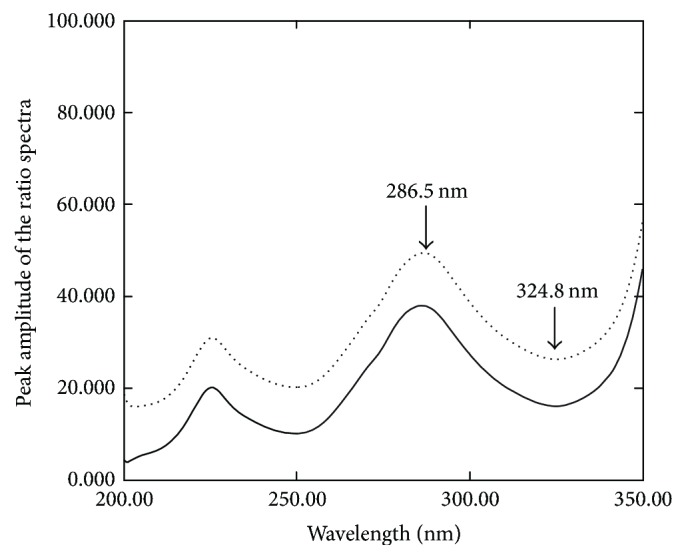
The ratio spectra of 10 *µ*g/mL OXY (—) and laboratory prepared mixture containing 10 *µ*g/mL FLU and 10 *µ*g/mL OXY (⋯) using normalized spectra of FLU as a divisor.

**Figure 5:**
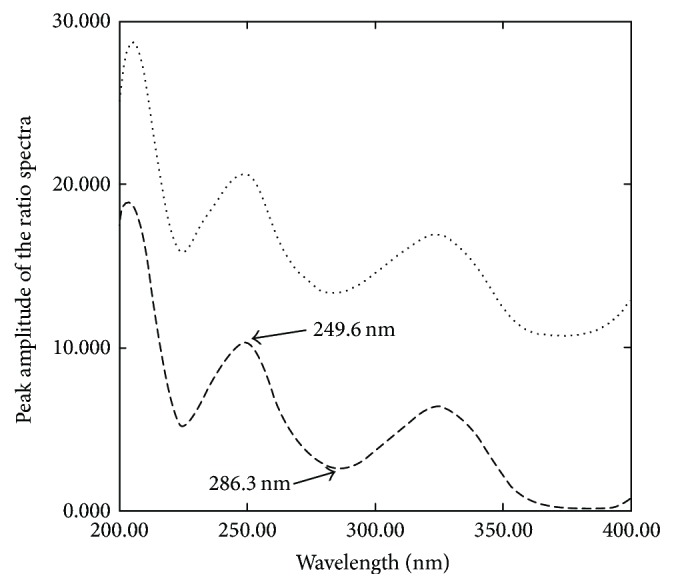
The ratio spectra of 10 *µ*g/mL FLU (- - - -) and laboratory prepared mixture containing 10 *µ*g/mL FLU and 10 *µ*g/mL OXY (⋯) using normalized spectra of OXY as a divisor.

A simple and selective technique with minimal data manipulation for the resolution of overlapped spectra by rapid calculation of the difference of the amplitude of the ratio spectra at appropriate wavelengths [29, 30, 33] was suggested as a third method for resolving the aforementioned drugs. The important factor in obtaining the ratio spectra is the choice of a good divisor. Different divisors including 60 *µ*g/mL and normalized divisor spectra of OXY and FLU were tried to obtain the ratio spectra of OXY and FLU. The best results regarding selectivity and baseline noise were obtained upon using normalized divisor spectra of OXY and FLU for determination of FLU and OXY, respectively. The used normalized spectra of OXY and FLU as divisors facilitate the optimization of the working conditions and diminish quantitation errors obtained by dividing the spectra for several standards of variable concentration into the corresponding concentration [34]. Different wavelength pairs were investigated to meet the method requirements. The difference of the amplitude of the ratio spectra at 286.5 nm–324.8 nm and 249.6 nm–286.3 nm was selected for the determination of OXY and FLU, respectively (Figures 4 and 5).

**Figure 6:**
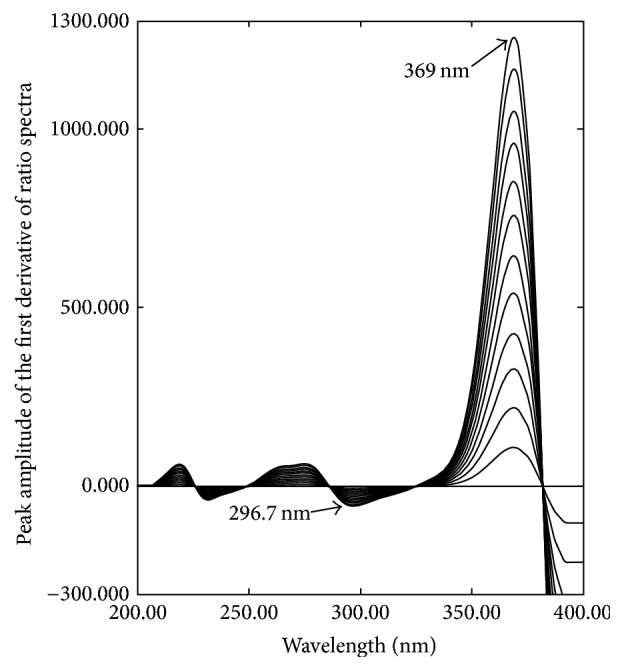
First derivative of ratio spectra of OXY (5–60 *µ*g/mL) using normalized divisor of FLU and 0.1 N HCl as a blank.

**Figure 7:**
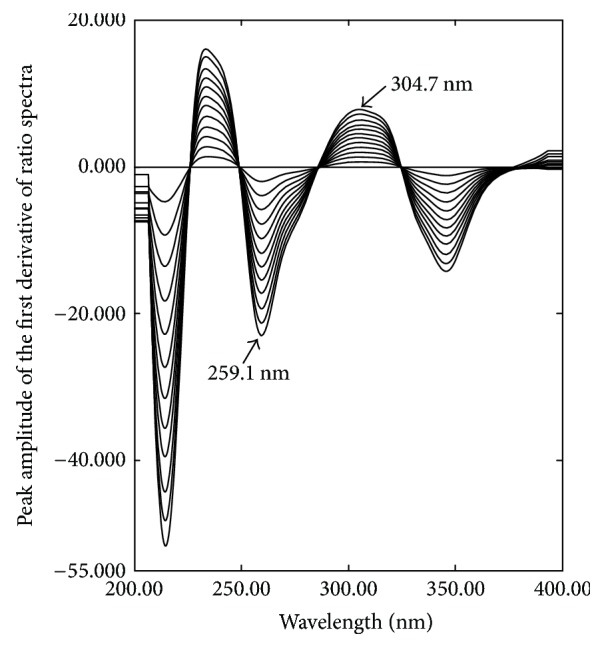
First derivative of ratio spectra of FLU (5–60 *µ*g/mL) using a normalized divisor of OXY and 0.1 N HCl as a blank.

The first derivative of ratio spectra spectrophotometric method (1DD) is a popular method for resolving a mixture of two interfering components [35]. Different parameters that affect the shape of the derivative of the ratio spectra such as wavelength, scanning speed, and the wavelength increment over which the derivative is obtained (Δ*λ*) were studied. It was found that fast scanning speed, Δ*λ* = 8, and scaling factor 10 give the best compromise in terms of signals to noise ratio, peak resolution, and sensitivity throughout the determinations. The obtained first derivative of the ratio spectra of OXY and FLU is shown in Figures 6 and 7, respectively. OXY can be successfully determined by measuring peak to peak maximum at 296.7 nm and 369 nm, while FLU can be successfully determined by measuring peak to peak maximum at 259.1 nm and 304.7 nm.

Linear relationships were obtained between the concentrations of OXY and FLU in the range of 5–60 *µ*g/mL and responses in each proposed spectrophotometric method. The regression equations and correlation coefficient were computed and shown in Table 1.

The stability of OXY and FLU in 0.1 N HCl has been studied by keeping samples of the drugs in tightly capped volumetric flasks, covered with aluminum foil and stored in the refrigerator. The samples were checked for assay in fourteen successive days of storage and compared with the freshly prepared sample. We found that the RSD values of the assay are below 2.0%, which indicates that both OXY and FLU are stable in their 0.1 N HCl solutions for 2 weeks.

### 4.1. Method Validation

Validation was done according to ICH guidelines [36]. Linearity, accuracy, range, and precision (repeatability and intermediate precision) were determined. Satisfactory results were obtained and illustrated in Table 1. Selectivity was also determined by applying the proposed methods for the determination of cited drugs in laboratory prepared mixtures containing different ratios of OXY and FLU (one of them represents the pharmaceutical formulation ratio). Good results are obtained indicating good selectivity of the proposed methods (Table 2).

The suggested methods are fruitfully applied for the determination of OXY and FLU in their pharmaceutical formulation. Satisfactory results indicate that there is no interference from dosage form excipients. Standard addition technique was applied to evaluate the accuracy of the developed methods for the analysis of drugs in the dosage form. The obtained results are listed in Tables 3 and 4.

Statistically comparison was done between the proposed methods and the reported direct spectrophotometric method for OXY [26] and official direct spectrophotometric method for FLU [5]. The obtained results show no significant difference between them (Table 5).

## 5. Conclusion

The proposed methods give the first contribution for the simultaneous determination of Oxytetracycline HCl and Flunixin Meglumine in the veterinary pharmaceutical dosage form. The developed spectrophotometric methods are validated and successfully applied for the simultaneous determination of OXY and FLU as a binary mixture in their pure form and in their available dosage form without any preliminary separation steps, which do not require complex algorithm, software programs (like Matlab), or sophisticated calculation. The proposed methods are simple, accurate, sensitive, selective, and reproducible and therefore can be applied for the routine work in QC laboratories of any poor country lacking liquid chromatographic instrument.

## Figures and Tables

**Table 1:** Assay validation sheet of the proposed methods for the determination of Oxytetracycline HCl and Flunixin Meglumine.

Parameter	Dual wavelength		First derivative		Ratio difference		Derivative ratio	
	OXY	FLU	OXY	FLU	OXY	FLU	OXY	FLU
*Accuracy* (mean ± RSD)	99.76 ± 1.00	100.36 ± 0.71	99.66 ± 0.98	100.50 ± 0.77	99.01 ± 0.89	100.01 ± 0.91	99.92 ± 1.11	100.04 ± 1.00
Repeatability precision ^*∗*^	99.87 ± 1.18	100.56 ± 0.87	99.83 ± 1.18	100.82 ± 0.81	99.40 ± 1.04	100.23 ± 0.94	99.70 ± 1.41	100.26 ± 1.15
Intermediate precision^*∗∗*^	100.47 ± 1.81	100.7 ± 0.97	100.84 ± 1.71	101.28 ± 0.43	100.79 ± 0.99	100.71 ± 1.34	101.37 ± 1.04	100.89 ± 1.42
*Linearity*								
Slope	0.0059	0.0059	0.0038	0.0023	0.7847	0.3142	1.3421	0.1583
Intercept	0.0139	0.0216	0.0072	0.0088	2.1503	0.7429	2.1807	0.5127
Correlation coefficient (*r*)	0.9999	0.9999	0.9998	0.9999	0.9999	0.9999	0.9998	0.9998
Range	7–60 *µ*g/mL	5–60 *µ*g/mL	5–60 *µ*g/mL	5–60 *µ*g/mL	5–60 *µ*g/mL	5–60 *µ*g/mL	5–60 *µ*g/mL	5–60 *µ*g/mL

^*∗*^The intraday (*n* = 3), RSD of three concentrations of 10, 30, and 60 *µ*g/mL for OXY and of 10, 30, and 60 *µ*g/mL for FLU repeated three times within the day. ^*∗∗*^The interday (*n* = 3), RSD of three concentrations of 10, 30, and 60 *µ*g/mL for OXY and of 10, 30, and 60 *µ*g/mL for FLU repeated three times in three successive days.

**Table 2:** Result obtained for the determination of Oxytetracycline HCl and Flunixin Meglumine in laboratory prepared mixtures by the proposed spectrophotometric methods.

Conc. of lab. mix. (*µ*g/mL)		Recovery % of OXY				Recovery % of FLU			
FLU	OXY	Dual WL	D^1^	ΔP	D R	Dual WL	D^1^	ΔP	DR
10	10	101.80	100.74	102.11	101.33	98.04	97.95	98.95	97.31
10	20	100.06	100.14	100.13	100.82	97.73	98.52	97.72	98.41
20	10	100.84	101.20	101.21	100.30	99.64	100.40	98.72	98.83
10	35	101.16	100.87	101.06	101.95	100.20	100.42	99.18	98.72
Mean		100.97	100.74	101.13	101.10	98.90	99.32	98.64	98.32
SD		0.72	0.44	0.81	0.71	1.20	1.28	0.64	0.70
RSD%		0.72	0.44	0.80	0.70	1.22	1.29	0.65	0.71

**Table 3:** Determination of Oxytetracycline HCl and Flunixin Meglumine in FLOXON by the proposed dual wavelength and first-derivative spectrophotometric methods and application of standard addition technique.

Method	Drug	Found (%)^*∗*^ ± SD	Standard addition technique		
			Pure added (*µ*g/mL)	Found (*µ*g/mL)	Recovery %
Dual wavelength	OXY	103.87 ± 1.75	5	5.07	101.40
			25	25.07	100.28
			30	30.40	101.33
	Mean ± RSD				101.00 ± 0.62
	FLU	97.14 ± 1.63	5	4.99	99.80
			10	10.08	100.80
			15	15.28	101.87
	Mean ± RSD				100.82 ± 1.03

First derivative	OXY	103.76 ± 0.58	5	5.03	100.60
			25	24.89	99.56
			30	30.03	100.10
	Mean ± RSD				100.09 ± 0.52
	FLU	98.07 ± 1.00	5	4.91	98.20
			10	9.81	98.10
			15	14.85	99.00
	Mean ± SD				98.43 ± 0.50

^*∗*^Average of three determinations of FLOXON tables (labeled to contain 108 mg/mL Oxytetracycline HCl and 33 mg/mL Flunixin Meglumine), BN 160142.

**Table 4:** Determination of Oxytetracycline HCl and Flunixin Meglumine in FLOXON by the proposed ratio difference and first derivative of ratio spectra spectrophotometric methods and application of standard addition technique.

Method	Drug	Found (%)^*∗*^ ± SD	Standard addition technique		
			Pure added (*µ*g/mL)	Found (*µ*g/mL)	Recovery %
Ratio difference	OXY	102.40 ± 1.06	5	4.95	99.00
			25	24.82	99.28
			30	29.93	99.77
	Mean ± RSD				99.35 ± 0.39
	FLU	97.50 ± 1.23	5	4.92	98.40
			10	10.04	100.40
			15	15.13	100.87
	Mean ± RSD				99.89 ± 1.31

Derivative ratio	OXY	102.74 ± 1.07	5	5.09	101.80
			25	25.31	101.24
			30	30.55	101.83
	Mean ± RSD				101.62 ± 0.33
	FLU	96.63 ± 1.43	5	5.01	100.20
			10	10.09	100.90
			15	15.12	100.80
	Mean ± SD				100.63 ± 0.38

^*∗*^Average of three determinations of FLOXON tables (labeled to contain 108 mg/mL Oxytetracycline HCl and 33 mg/mL Flunixin Meglumine), BN 160142.

**Table 5:** Statistical comparison of the results obtained by the proposed spectrophotometric methods and the reported method for the determination of Oxytetracycline HCl [26] or official method for the determination of Flunixin Meglumine [5] in their pure form, respectively.

Items	Oxytetracycline HCl					Flunixin Meglumine				
	Dual WL	D1	ΔP	D R	Reported method^a^	Dual WL	D1	ΔP	D R	Official method^b^
Mean	99.76	99.66	99.01	99.92	99.16	100.36	100.50	100.01	100.04	99.84
SD	1.00	0.98	0.89	1.11	0.89	0.71	0.77	0.91	1.00	0.89
Variance	1.00	0.96	0.79	1.23	0.79	0.50	0.59	0.82	1.00	0.79
*n*	5	5	5	5	5	5	5	5	5	5
Student's *t*-test (2.31)^*∗*^	1.00	0.85	0.27	1.20		1.02	1.26	0.30	0.33	
*F* test (6.39)^*∗*^	1.27	1.22	1.00	1.57		1.58	1.34	1.04	1.27	

^a^Zero-order spectrophotometric method. ^b^Zero-order spectrophotometric method. ^*∗*^The values in the parenthesis are the corresponding theoretical values of *t* and *F* at *p* = 0.05.
